# Epidemiological investigation and management of bloody diarrhea among children in India

**DOI:** 10.1371/journal.pone.0222208

**Published:** 2019-09-13

**Authors:** Rahul Bawankule, Sadanand Shetye, Ashish Singh, Abhishek Singh, Kaushalendra Kumar

**Affiliations:** 1 International Institute for Population Sciences, Mumbai, India; 2 B. K. L. Walawalkar Hospital and Rural Medical College, Kasarwadi-Sawarde, India; 3 SJM School of Management, Indian Institute of Technology Bombay, Powai, Mumbai, India; Institute of Economic Growth, INDIA

## Abstract

**Background:**

The evidence on the factors associated with childhood bloody diarrhea in developing countries in general and India, in particular, is somewhat limited. Our study, therefore, examines—the prevalence of bloody diarrhea; the magnitude of treatment of bloody diarrhea (use of both oral rehydration and antibiotics (pills, syrups, and injections)); and several other associated factors with bloody diarrhea in the youngest children under five years in the Indian context.

**Methods:**

We used data from the National Family Health Survey (NFHS)—4 conducted in 2015–16. We used a multivariable binary logistic regression model to identify the factors associated with bloody diarrhea. We also applied a multinomial logistic regression model to identify associated factors with the treatment of bloody diarrhea amongst the youngest children below five years.

**Findings:**

The overall prevalence of bloody diarrhea in the youngest children was about 9 percent in the last two weeks preceding the survey. There was a significant difference in the mean age of those children having bloody diarrhea and watery diarrhea during the same period. Children whose stools were disposed of unsafely and those who belonged to households with neither a place nor water for washing hands were more likely to suffer from bloody diarrhea compared to their counterparts with these facilities. About a little less than one-fifth of the youngest children (16%) received adequate treatment of bloody diarrhea. The treatment of bloody diarrhea was associated with the health facility and maternal and children’s socioeconomic and demographic characteristics.

**Conclusion:**

The study shows that household environmental risk factors are important predictors of bloody diarrhea amongst the youngest children. Still, 28% of those children did not receive any treatment of bloody diarrhea in India. There is also a clear need to promote the practice of safe disposal of children’s stools and handwashing among mothers and children. Mothers need to be sensitized about the necessity of an immediate visit to a health facility/center in case of bloody diarrhea.

## Introduction

Diarrhea is a major public health concern in underdeveloped and developing countries, even though it can be prevented and treated with simple measures. It is a typical manifestation of gastrointestinal infections caused by a wide range of pathogens including bacteria, viruses, and protozoa [1]. Despite a significant decline in diarrheal deaths in under-five children globally (from 1.2 million in 2000 to 0.5 million in 2016), it is still the second leading cause of under-five mortality in the world [2]. A recent estimate suggests that diarrhea kills more than 1,400 under-five children every day worldwide, and was responsible for 9 percent of child deaths in 2015 [3]. Among the countries, India has the highest burden of diarrheal deaths in children under the age of five, and there were nearly 102,813 under-five deaths in 2016 alone [2].

Having said that, there are four clinical types of diarrhea: acute watery diarrhea (including cholera), acute bloody diarrhea (also called dysentery), persistent diarrhea and diarrhea with severe malnutrition (marasmus or kwashiorkor) [4]. Bloody diarrhea refers to any diarrheal episode in which the loose or watery stool contains visible red blood. It is a sign of invasive enteric infection that carries a significant risk of severe morbidity and death in young children especially in underdeveloped and developing countries [5]. Bloody diarrhea is associated with intestinal damage and nutrient deterioration in children [6]. The duration of bloody diarrhea is longer and generally associated with more complications than watery diarrhea. It affects the child’s growth adversely and has a high case fatality rate. Existing statistics indicate that nearly 10 percent of the total diarrheal episodes globally have visible blood in the stools [7]. A recent study reported that acute bloody diarrhea accounted for 5–15% of all diarrheal deaths in children aged 0–59 months in seven low and middle-income countries (Bangladesh, Ethiopia, Ghana, India, Pakistan, Uganda, and the United Republic of Tanzania) [8].

Focusing on underdeveloped and developing countries; maximum episodes of bloody diarrhea are in young children resulting from an enteric infection caused by invasive bacteria. The nonbacterial pathogens account for less than 3 percent of episodes of bloody diarrhea among these countries. In most cases, the invasive bacterial infection is transmitted by excreta of the infected individual through direct fecal-oral contamination. The greatest incidence of bloody diarrhea is seen at the time of weaning when children learn to walk and crawl and are introduced to solid food. The increased mobility of children enhances susceptibility to fecal pathogens [5]. Inadequate sanitation, overcrowding, contaminated food, and use of public water system expose children to the risk of invasive bacterial infections [9–12].

Though there are a few studies which have identified the socioeconomic, demographic and environmental factors associated with bloody diarrhea, the scientific scholarship on the subject is still limited. A study in Turkey reported that children who suffered from bloody diarrhea were on an average, older compared to children who suffered from watery diarrhea [13]. While separate storage of drinking water and water for washing hands after the last defecation were protective against bloody diarrhea, the contaminated source of water was a risk factor in Kenya [14]. Another study from Kenya found that sharing toilet facilities by multiple families was associated with bloody diarrhea [15]. In Iraq, the prevalence of bloody diarrhea was significantly higher among children in the age group 1–3 years, those residing in rural areas, those with illiterate mothers, and those who were exclusively on bottle feeding [7].

Acute bloody diarrhea is an indication of a life-threatening disorder and needs immediate treatment and care [16]. Early medical care prevents the risk of non-response to medical treatment in severe conditions [17]. The Integrated Management of Childhood Illness (IMCI) guidelines recommend immediate referral of children with bloody diarrhea to health facility/center and should be managed with antibiotics, fluids, feeding, and follow up [18]. In the Global Enteric Multicenter Study (GEMS), about 50 percent of the caregivers considered bloody diarrhea as the most dangerous form of diarrhea [19]. In Mexico, mothers identified bloody diarrhea as a clinical sign of the worsening condition of the child and sought timely medical assistance when compared to mothers whose children had acute diarrhea [20]. A study by Fissehaye et al. (2018) revealed that blood in stools was one of the significantly associated factors with maternal health-seeking behavior for childhood diarrhea [21] Other studies from Turkey and Bangladesh found that caregivers of children suffering from bloody diarrhea visited a hospital immediately when compared to those whose children suffered from watery diarrhea [13, 22].

Given the significant and substantial contribution of bloody diarrhea to childhood diarrheal morbidity and mortality in underdeveloped and developing countries, it is necessary to examine its epidemiology and treatment seeking in large and socioeconomically diverse countries like India. In India, open defecation and overcrowding are very common. The burden of under-five deaths associated with diarrhea is also the highest in India [3, 23]. Further, the mother/caregiver’s health care seeking behavior is often affected by socioeconomic and demographic factors, accessibility and availability of health services [24]. While a number of studies have investigated treatment seeking for childhood watery diarrhea, no study (to the best of our knowledge) has so far identified factors affecting mother/caregiver’s health-seeking behavior for bloody diarrhea in children or has identified the socioeconomic and environmental factors that are associated with bloody diarrhea and treatment seeking in India. Hence, the objective of this study is to identify the associated factors with bloody diarrhea in children in India. The study also aims to determine the magnitude and associated factors of the treatment of bloody diarrhea (use of both antibiotics pills, syrups, and injections (hereafter antibiotics) and oral rehydration) in children below five years of age using a large-scale, population-based nationally representative data in India.

## Methods

### Data source

We used data from the most recent round of NFHS conducted in 29 states and 6 Union Territories in 2015–16 in India. The NFHS-4 is a nationally representative population-based cross-sectional household survey. The primary objective of NFHS-4 is to provide essential data on health and family welfare, as well as data on emerging issues like non-communicable diseases, menstrual hygiene, risk factors for non-communicable diseases. The NFHS-4 adopted a stratified two-stage sampling design in rural and urban areas for selecting households and eligible men and women for an interview. The survey interviewed 601,509 households, 699,686 women aged 15–49, and 112,122 men aged 15–54. The response rates for household, women, and men were 98 percent, 97 percent, and 92 percent, respectively. More details about the survey can be obtained from the publicly available India NFHS-4 report [25].

### Outcome variable

We included two outcome variables in the study. The first outcome variable is the occurrence of bloody diarrhea. For assessing the occurrence of bloody diarrhea in children, NFHS-4 asked two questions to mothers of children under-five. First, ‘Has (NAME) had diarrhea in the last two weeks?’ If mothers said ‘yes,’ they were further questioned, ‘Was there any blood in the stools?’ Hence, the outcome variable bloody diarrhea is a binary variable and is coded as `0' if there was no blood in the stools and `1' otherwise.

We generated the second outcome variable ‘treatment of bloody diarrhea’ based on IMCI guidelines for management of bloody diarrhea [18]. The variable was generated using the information about the use of ‘oral rehydration’ and ‘antibiotic pills, syrups, and injections’ for treating diarrhea. In NFHS-4, mothers were asked if children had diarrhea/bloody diarrhea ‘What (else) was given to treat the diarrhea?’ Hence, the second outcome variable is ‘treatment of bloody diarrhea,’ and it has three categories. The variable is coded as ‘0’ if children received neither oral rehydration nor antibiotic (no treatment), coded as ‘1’ if the children received either oral rehydration or antibiotic (inadequate treatment) and coded as ‘2’ if received both oral rehydration and antibiotic (adequate treatment).

### Exposure variables

We included a number of household environmental risk factors as exposure variables in the analysis based on previous studies reporting the linkages between household sanitation and drinking water source and occurrence of bloody diarrhea in children [14, 15]. We included the type of toilet facility, drinking water source and treatment, disposal of children’s stools, unsafe disposal of children’s stools in the neighborhood and the provision of a place to wash hands in the households as exposure variables. We categorized the type of toilet facility and source of drinking water as ‘improved’ and ‘unimproved.’ We further categorized the source of drinking water as ‘improved and treated,’ ‘improved and untreated,’ ‘unimproved and treated’ and ‘unimproved and untreated’ [25]. We categorized the disposal of children’s stool into ‘safe’ and ‘unsafe’ by following WHO/UNICEF definition [26]. In the NFHS-4, the mother was asked: “The last time (NAME OF YOUNGEST CHILD) passed stools, what was done to dispose of the stools”? If the mother put/rinsed child’s last stools into a toilet or latrine, buried, or the child used a latrine or toilet, disposal of children’s stools was categorized as ‘safe.’ It was categorized as ‘unsafe’ if mother put/rinsed child’s last stools into drain/ditch, threw into the garbage or left into the open.

Our analysis also includes a variable on unsafe disposal of children’s stools in the neighborhood. First, we counted all children other than the index child whose stools disposal information was available in the primary sampling unit (PSU). We also summed all children excluding index child whose stools were disposed off unsafely within same PSU. Finally, we constructed the variable ‘unsafe disposal of children’s stool in the neighborhood’ by taking the proportion of unsafe disposal of children’s stools for all children other than the index child over disposal of children’s stools (other than index child) in the primary sampling unit (PSU). The NFHS-4 is the first large-scale population-based nationally representative survey that collected information on the provision of a dedicated place for washing hands in the households. We categorized it as ‘provision of a place for washing hands with both water and cleansing agent (soap/detergent (bar, liquid, powder, paste), ash, mud, and sand)),’ ‘provision of a place with water to wash hands’ and ‘lack of a place to wash hands.’

### Control variables

The previous studies indicated that age of child, place of residence, literacy status of mother and bottle feeding are significant risk factors of bloody diarrhea and so we included these variables as control variables in the analysis [7, 13]. We included few other socioeconomic and demographic factors causing watery diarrhea as control variables for bloody diarrhea given the limited evidence in the literature about their role in the causation of bloody diarrhea. The control variables included in the analysis of (risk of) occurrence of bloody diarrhea are ‘initiation of breastfeeding (within 1 hour, within 1–24 hours, after 24 hours) [27], bottle feeding (yes, no), age of child (below 12 months, 12–23, 24–35, 36–47, 48–59 months) [7], sex of child (male, female), size of child at birth (smaller than average, average and larger) [23]. The analysis also includes literacy of mother (literate, non-literate) [7], exposure to mass media (exposed, unexposed) [28], religion (Hindu, other) [23], caste/tribe (Scheduled Caste (SC) and Scheduled Tribe (ST), other) [29], household wealth status (poor, middle, rich) [28], and place of residence (urban, rural) [7] as control variables.

The control variables for ‘treatment of bloody diarrhea’ are first place of treatment sought for bloody diarrhea (public, private and other) [30], days within treatment sought after onset of bloody diarrhea (same day, 1–2 days, 2+ days) [31], age of child (below 18 months, 18–35 months, 36–59 months) [30], sex of child (male, female) [30], literacy of mother (literate, non-literate) [30], exposure to mass media (exposed, unexposed) [32], religion (Hindu, other) [33], caste/tribe (SC and ST, other) [34], household wealth status (poor, middle, rich) [35], and place of residence (urban, rural) [32]. The detail descriptions of the exposure and control variables can be seen in S1 File.

In NFHS-4, the household wealth index is computed using principal component analysis by assigning weights to a number of household assets. The household wealth index also includes ‘type of toilet facility’ and ‘source of drinking water.’ Since we wanted to examine the independent effect of ‘type of toilet facility’ and ‘source of drinking water’ on the occurrence of bloody diarrhea, we computed a new wealth index that did not include these two variables. We followed the procedure of NFHS-4 to construct a new wealth index and categorized households into the lowest one-third (poor), middle one-third (middle) and the highest one-third (rich). Mother’s exposure to mass media includes exposure to any source of mass media: newspaper, radio, and television. Mothers exposed to at least one of the sources of mass media were coded as ‘exposed.’ The rest were coded as ‘unexposed.’

### Inclusion criteria

In NFHS-4, the information on bloody diarrhea was collected for the last three children born during the five years preceding the survey. But the questions related to children’s stool disposal and initiation of breastfeeding after birth were asked with respect to the youngest child born in the five years preceding NFHS-4. Hence, we included only the youngest child in the analysis. The final sample size for our study is 19,490 children.

### Statistical analysis

We estimated multivariable binary logistic regression model to identify the socioeconomic, demographic and household environmental risk factors of bloody diarrhea. We applied a multinomial logistic regression model to identify the socioeconomic and demographic factors associated with the treatment of bloody diarrhea in children.

The analysis was carried out in STATA 13.0. Appropriate sampling weights were used in the estimations to take into account the complex survey design of NFHS-4. All the independent variables were tested for possible multicollinearity before putting those into the regression models.

### Ethical approval

Our study is based on a publicly available dataset, Demographic and Health Survey (DHS) (also known as the National Family Health Survey (NFHS) in India). It does not have any identifiable information of the survey participants. One can access the data from the DHS website https://dhsprogram.com/data/dataset/India_Standard-DHS_2015.cfm?flag=0 after obtaining permission from the DHS. The authors did not have special privileges other interested researchers would not have. DHS followed all the ethical protocols strictly, including informed consent. Hence, no ethical approval is required for the current study.

## Results

**Fig 1:**
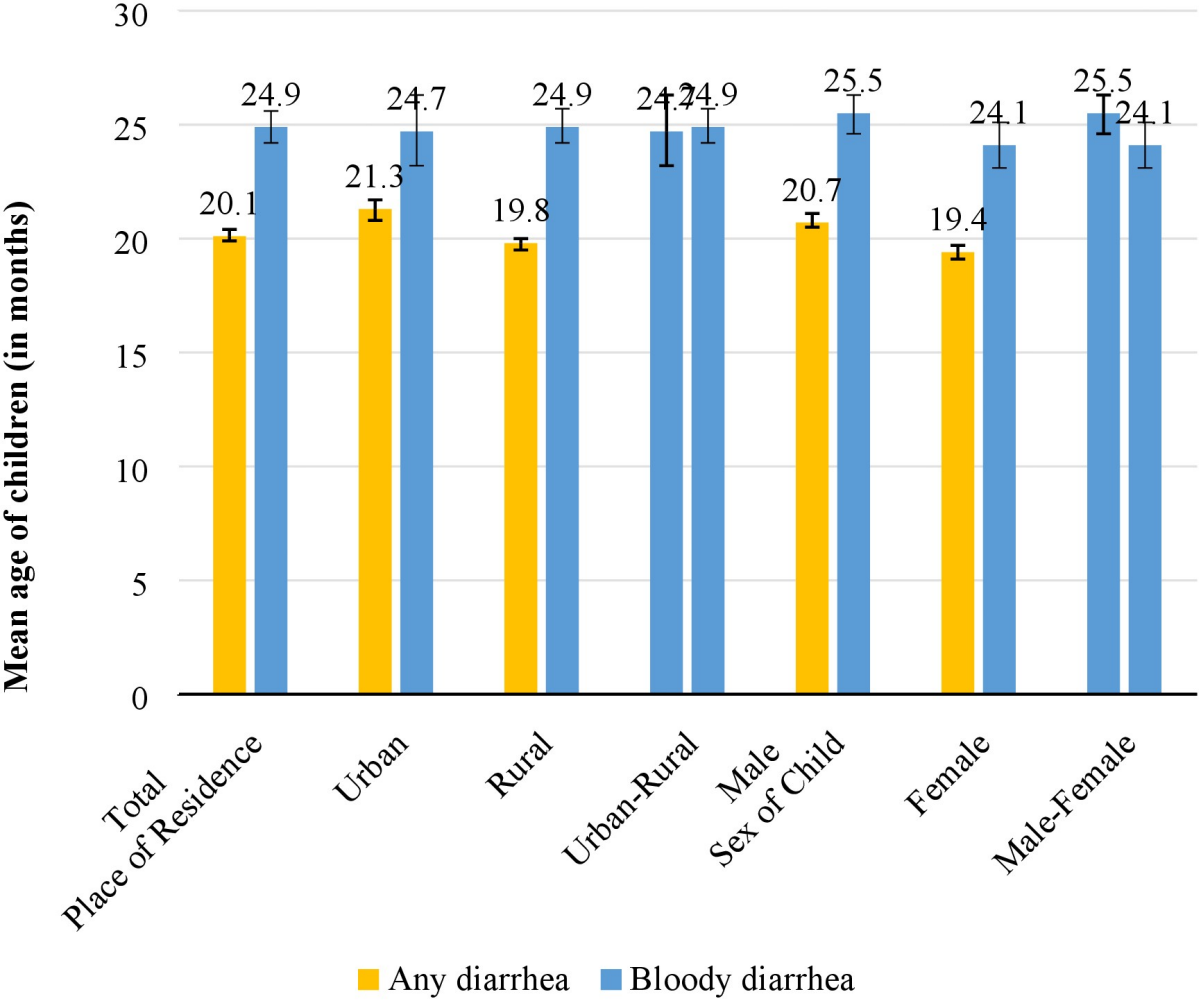
Mean age of children below five years who suffered from bloody diarrhea by place of residence and sex of child, India, 2015–16.

The mean age of children who suffered from bloody diarrhea (25 months; SD ±15) was statistically higher than the mean age of children who suffered from watery diarrhea (20 months; SD ±20). Also, the mean age of children who suffered from bloody diarrhea was higher for male children (25 months; SD ±15) than for female children (24 months; SD ±15). Further, the mean age for the occurrence of bloody diarrhea in children did not vary by place of residence (Fig 1).

**Fig 2:**
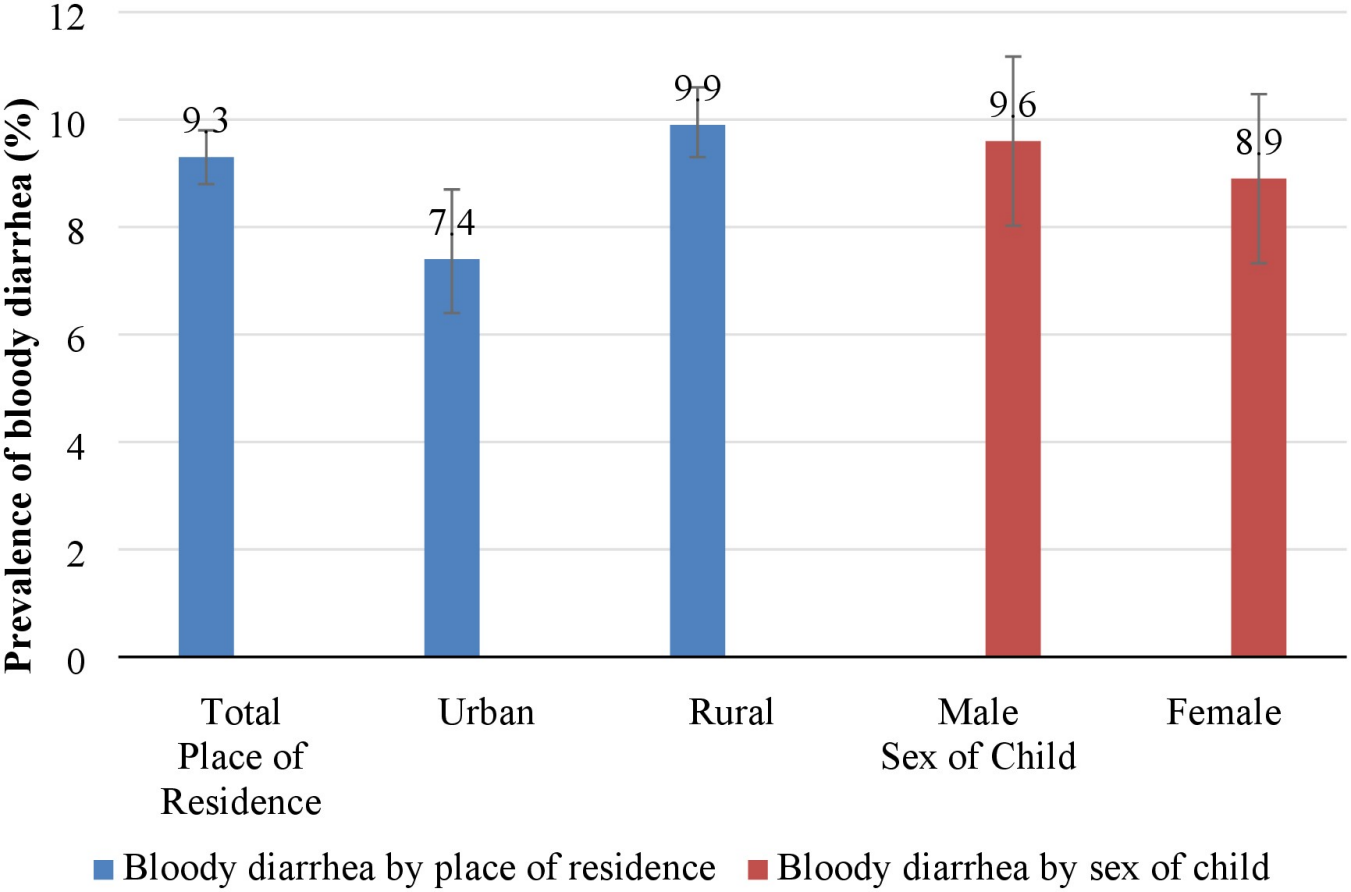
Prevalence of bloody diarrhea in children below five years by place of residence and sex of child, India, 2015–16.

A little less than one-tenth of the children under-five suffered from bloody diarrhea during the two-weeks preceding NFHS-4. The prevalence of bloody diarrhea varied considerably by place of residence—it was higher among children residing in rural areas (10%; 95% CI: 8.8–9.8%) compared with children in urban areas (7%; 95% CI: 6.4–8.7%). Prevalence of bloody diarrhea did not vary by the sex of the children (Fig 2).

**Fig 3:**
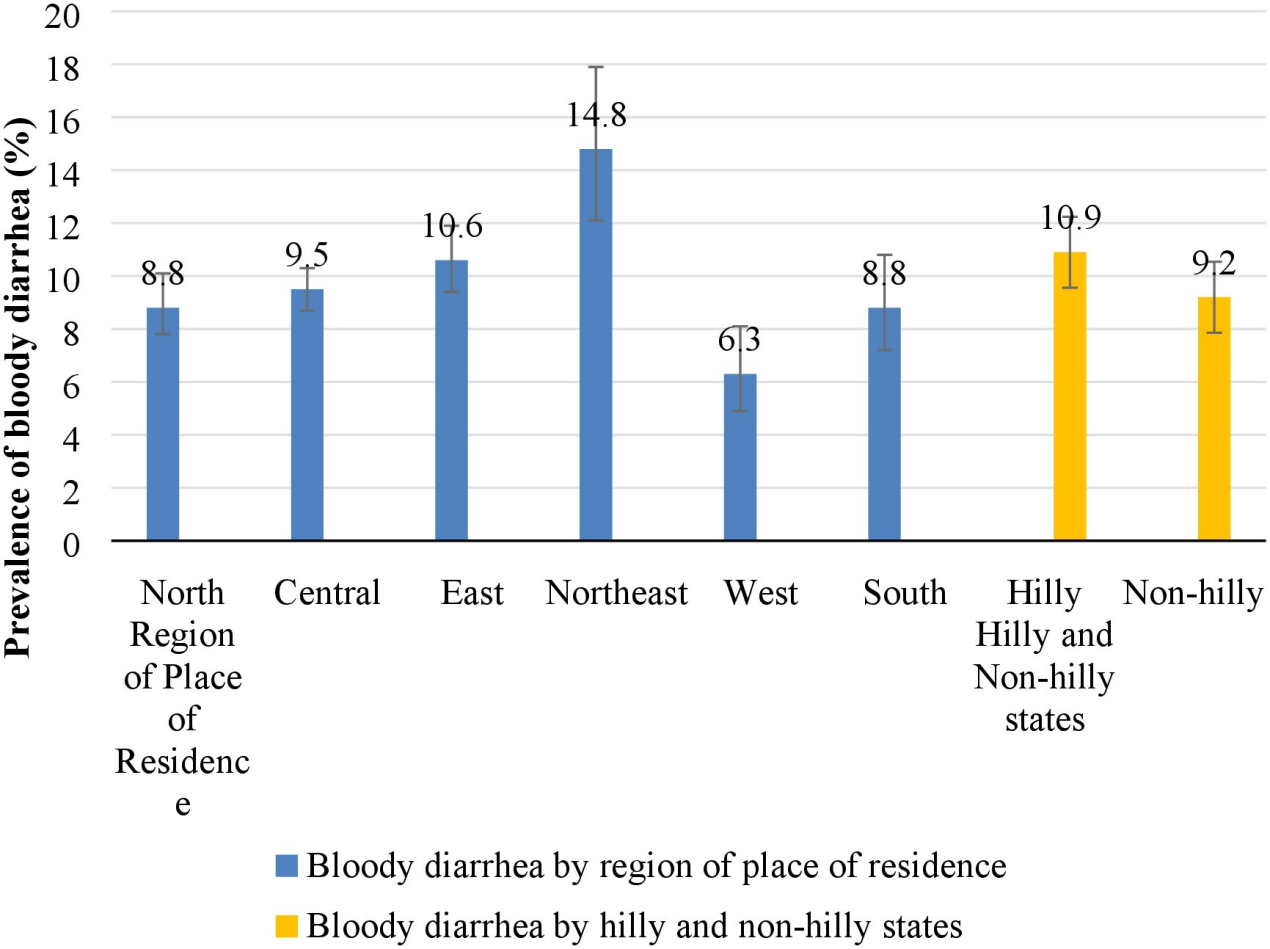
Prevalence of bloody diarrhea in children below five years by region of place of residence and hilly and non-hilly states, India, 2015–16.

The prevalence of bloody diarrhea also varied significantly with regions of the place of residence and hilly and non-hilly states in India. The highest prevalence of bloody diarrhea was reported in children belonging to Northeast region (15%; 95% CI: 12.1–17.9%) followed by East region (11%; 95% CI: 9.4–11.9%) while it was lowest in West region (6%; 95% CI: 4.9–8.1%). The bloody diarrhea was higher among children residing in hilly states (11%; 95% CI: 9.4–12.5%) compared to children in non-hilly states (9%; 95% CI: 8.7–9.8%) (Fig 3).

The prevalence of bloody diarrhea in children by socioeconomic, demographic and household environment related variables in children is shown in Table 1. Bloody diarrhea was higher among children who belonged to households with unimproved toilet facility (10% vs. 8%). The prevalence of bloody diarrhea was also higher among children from households using untreated drinking water (9–10%). The bloody diarrhea was higher among children whose stools were disposed off unsafely (10%) compared with children whose stools were disposed off safely (8%). The prevalence of bloody diarrhea was more prevalent in households that had neither a dedicated place for washing hands nor water for hand wash (12%).

**Table 1:** Prevalence of bloody diarrhea^1^ in children below five years by socioeconomic, demographic and household environment related variables, India, 2015–16.

Socioeconomic, demographic and household environment related Variables	Prevalence (95% CI)	Number (N)
**Type of toilet facility**		
Improved	7.8 (7.0–8.8)	6262
Unimproved	10.1 (9.4–10.7)	12728
**Drinking water source and treatment**		
Improved and treated	7.9 (6.9–9.0)	4505
Improved and untreated	10.1 (9.4–10.9)	11956
Unimproved and treated	6.0 (4.6–7.8)	1033
Unimproved and untreated	9.0 (7.5–10.7)	1996
**Children’s stools disposal**		
Safe	8.0 (7.1–9.0)	6886
Unsafe	10.0 (9.3–10.7)	12563
**Presence of handwashing place**		
With both water and cleansing agent	7.9 (7.3–8.6)	10823
With water only	10.0 (9.0–11.1)	5239
Neither handwashing place nor water for hand wash	12.4 (11.0–14.0)	3428
**Initiation of breastfeeding after birth**		
Within 1 hour	9.5 (8.6–10.5)	6935
Within 1–24 hour	8.6 (7.9–9.5)	8106
After 24 hours	9.2 (8.1–10.5)	3705
**Bottle feeding**		
No	9.1 (8.5–9.7)	15204
Yes	10.0 (8.8–11.2)	4286
**Age of child (in months)**		
below 12 months	5.7 (5.0–6.5)	6523
12–23 months	9.8 (8.9–10.9)	6288
24–35 months	10.9 (9.6–12.3)	3313
36–47 months	12.7 (10.9–14.8)	2023
48–59 months	15.0 (12.7–17.5)	1343
**Size of child at birth**		
Smaller than average	9.1 (7.9–10.4)	3082
Average and larger	9.3 (8.7–9.9)	16408
**Literacy status of mother**		
Literate	8.2 (7.6–8.8)	13746
Non-literate	11.9 (10.9–13.0)	5744
**Mother exposed to mass media**		
Exposed	8.6 (8.0–9.2)	14094
Unexposed	11.1 (10.1–12.2)	5396
**Religion**		
Hindu	9.0 (8.3–9.5)	15277
Other	10.5 (9.3–11.9)	4213
**Caste/Tribe**		
Other	8.1 (7.5–8.8)	12639
SC and ST	11.1 (10.1–12.1)	6233
**Household wealth status**		
Rich	7.4 (6.4–8.4)	5800
Middle	8.2 (7.4–9.1)	6953
Poor	12.1 (11.1–13.1)	6737
**Total**	**9.3 (8.8–9.8)**	**19490**

Abbreviations: CI, confidence interval; SC, scheduled caste; ST, scheduled tribe.

All values are weighted in the table and represent absolute numbers and percentages unless otherwise stated.

^1^ Estimated per 100 children with diarrhea.

**Fig 4:**
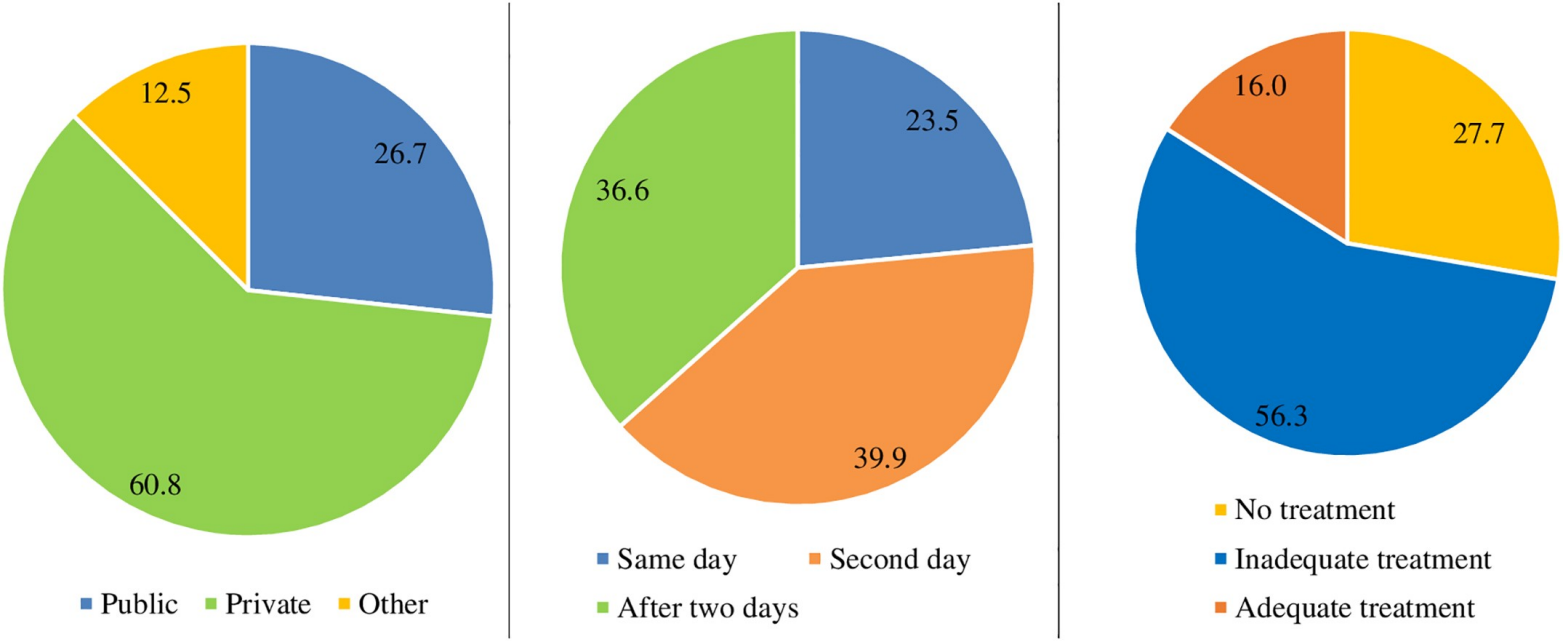
Figs 4a, 4b, and 4c. First place of treatment sought for bloody diarrhea, days within treatment sought after onset of bloody diarrhea, and treatment of bloody diarrhea, in children below five years, India, 2015–16.

The private health facility was the first place for three-fifths of children (61%; 95% CI: 57.5–64.0%) followed by public health facility (27%; 95% CI: 23.9–29.8%) for seeking treatment of bloody diarrhea. Only a little less than one-fourth of the children (23%; 95% CI: 20.8–26.3%) received treatment on the same day of bloody diarrhea onset. Overall, 16 percent of children (95% CI: 13.6–18.8%) below five years, who had bloody diarrhea received adequate treatment while 28 percent of children (95% CI: 24.8–30.7%) did not receive any treatment for bloody diarrhea (Fig 4a, 4b and 4c).

The treatment of bloody diarrhea by socioeconomic, demographic and residence-related variables in children is presented in Table 2. The public health facility was the major source of adequate treatment (27%) for children who had bloody diarrhea. About a little less than two-fifth of the children received adequate treatment within two days after the onset of bloody diarrhea. The adequate treatment sought was higher among children aged 18–59 months (18%) compared to children below 18 months (14%). Adequate treatment was higher among female children (17%) than male children. The adequate treatment was lower among children of non-literate mothers (12%), and mothers who were not exposed to mass media (10%). The adequate treatment was also affected by both religion and place of residence, and it was higher among children belonging to other religion (18%) and residing in urban areas (21%). Further, adequate treatment increased with higher level of household wealth status and was highest among children belonging to rich households (21%).

**Table 2:** Treatment of bloody diarrhea^1^ in children below five years by socioeconomic, demographic and residence-related variables, India, 2015–16.

Socioeconomic, demographic and residence related variables	Adequate treatment	Number
	Percentage (95% CI)	
**First place of treatment sought for bloody diarrhea**		
Other	10.4 (6.3–16.7)	204
Private	12.5 (9.6–16.1)	995
Public	26.7 (21.6–32.6)	437
**Days within treatment sought after onset of bloody diarrhea**		
After two days	11.3 (8.6–14.9)	600
Same day	17.7 (12.5–24.6)	384
Second day	19.3 (15.3–24.1)	652
**Age of child (in months)**		
Below 18 months	13.9 (10.8–17.7)	597
18–35	17.1 (13.2–21.8)	641
36–59	17.6 (13.3–22.8)	398
**Sex of child**		
Male	14.9 (12.3–18.0)	928
Female	17.5 (13.8–22.0)	708
**Literacy status of mother**		
Non-literate	12.1 (9.1–15.8)	606
Literate	18.3 (15.0–22.2)	1030
**Mother exposed to mass media**		
Unexposed	10.3 (6.9–15.0)	530
Exposed	18.8 (15.7–22.3)	1106
**Religion**		
Hindu	15.2 (12.7–18.1)	1229
Other	18.5 (12.9–25.9)	407
**Caste/Tribe**		
SC and ST	14.7 (11.2–19.1)	609
Other	14.9 (11.9–18.6)	936
**Household wealth status**		
Poor	13.3 (10.0–17.3)	722
Middle	16.4 (12.9–20.7)	535
Rich	20.8 (14.9–28.1)	379
**Place of residence**		
Rural	14.6 (12.2–17.4)	1281
Urban	21.2 (14.7–29.6)	355
**Total**	**16.0 (13.6–18.8)**	**1636**

Abbreviations: CI, confidence interval; SC, scheduled caste; ST, scheduled tribe.

All values are weighted in the table and represent absolute numbers and percentages unless otherwise stated.

^1^ Estimated per 100 children with bloody diarrhea.

The results of multivariable binary logistic regression showing the risk of bloody diarrhea in children under-five are shown in Table 3. We estimated multivariable binary logistic regression models to identify socioeconomic, demographic and household environmental risk factors of bloody diarrhea. The first model includes the type of toilet facility, drinking water source and treatment, children’s stool disposal and the presence of a place to wash hands in the households and socioeconomic, demographic characteristics related to mother and children. Among the household environmental risk factors in the first model, children’s stool disposal and the presence of a place to wash hands in the households were statistically associated with the risk of bloody diarrhea in children. Children whose stools were disposed off unsafely were 1.18 (AOR: 1.18; 95% CI: 1.05, 1.35) times as likely to suffer from bloody diarrhea as children whose stools were disposed off safely.

**Table 3:** Results of multivariable binary logistic regression showing the risk of bloody diarrhea in children below five years, India, 2015–16 (N = 18143).

Socioeconomic, demographic and household environmental related variables	AOR (95% CI)	
	Model 1	Model 2
**Type of toilet facility**		
Improved	Ref.	Ref.
Unimproved	1.06 (0.94–1.21)	1.05 (0.92–1.19)
**Drinking water source and treatment**		
Improved and treated	Ref.	Ref.
Improved and untreated	1.02 (0.89–1.16)	1.01 (0.89–1.15)
Unimproved and treated	0.93 (0.74–1.18)	0.93 (0.74–1.18)
Unimproved and untreated	1.11 (0.92–1.34)	1.10 (0.91–1.33)
**Children’s stools disposal**		
Safe	Ref.	Ref.
Unsafe	**1.18 (1.05–1.34)**	1.08 (0.93–1.25)
**Unsafe disposal of children’s stool in the neighborhood (%)**	**-**	**1.28 (1.04–1.57)**
**Presence of handwashing place**		
With both water and cleansing agent	Ref.	Ref.
With water only	0.98 (0.86–1.12)	0.98 (0.86–1.11)
Neither handwashing place nor water for hand wash	**1.16 (1.01–1.33)**	**1.15 (1.01–1.32)**
**Initiation of breastfeeding after birth**		
After 24 hours	Ref.	Ref.
Within 1 hour	1.12 (0.97–1.29)	1.12 (0.97–1.29)
Within 1–24 hour	1.03 (0.89–1.18)	1.03 (0.89–1.18)
**Bottle feeding**		
No	Ref.	Ref.
Yes	**1.27 (1.12–1.43)**	**1.27 (1.13–1.44)**
**Age of child (in months)**		
Below 12 months	Ref.	Ref.
12–23 months	**1.83 (1.60–2.10)**	**1.83 (1.60–2.10)**
24–35 months	**2.09 (1.79–2.44)**	**2.09 (1.79–2.44)**
36–47 months	**2.41 (2.03–2.88)**	**2.41 (2.41–2.88)**
48–59 months	**2.78 (2.29–3.38)**	**2.78 (2.29–3.38)**
**Sex of child**		
Female	Ref.	Ref.
Male	**1.11 (1.01–1.23)**	**1.11 (1.01–1.23)**
**Size of child at birth**		
Smaller than average	Ref.	Ref.
Average and larger	**1.17 (1.02–1.35)**	**1.17 (1.02–1.35)**
**Literacy status of mother**		
Literate	Ref.	Ref.
Non-literate	**1.24 (1.10–1.39)**	**1.23 (1.10–1.39)**
**Mother exposed to mass media**		
Unexposed	Ref.	Ref.
Exposed	1.07 (0.95–1.22)	1.08 (0.95–1.23)
**Religion**		
Hindu	Ref.	Ref.
Other	**1.19 (1.06–1.34)**	**1.22 (1.08–1.37)**
**Caste/Tribe**		
Other	Ref.	Ref.
SC and ST	**1.23 (1.10–1.36)**	**1.23 (1.11–1.37)**
**Household wealth status**		
Rich	Ref.	Ref.
Poor	**1.20 (1.01–1.43)**	1.18 (0.99–1.40)
Middle	1.04 (0.90–1.21)	1.03 (0.89–1.20)
**Place of residence**		
Urban	Ref.	Ref.
Rural	**1.19 (1.04–1.37)**	**1.15 (1.01–1.33)**

Abbreviations: AOR, adjusted odds ratio; CI, confidence interval; Ref, reference; SC, scheduled caste; ST, scheduled tribe.

Bold AOR indicates significant finding at p-value < 0.05.

The second model includes unsafe disposal of children’s stools in the neighborhood in addition to variables included in the first model. The results indicate a statistically significant association between unsafe disposal of children’s stool in the neighborhood and bloody diarrhea in children. A unit increase in the proportion of unsafe disposal of children’s stools in the neighborhood was associated with a 28 percent (AOR: 1.28; 95% CI: 1.04, 1.57) higher risk of bloody diarrhea in children. Moreover, children who belonged to households that did not have a place or water to wash hands were 1.15 (AOR: 1.15; 95% CI: 1.01, 1.32) times as likely to suffer from bloody diarrhea as children who belonged to households with a place with water for washing hands and a cleansing agent.

A number of socioeconomic and demographic variables were statistically associated with bloody diarrhea in children. Bottle feeding was statistically associated with a higher risk of bloody diarrhea in children. Bottle-fed children were 1.27 (AOR: 1.27; 95% CI: 1.13, 1.44) times as likely as children who were not bottle-fed to suffer from bloody diarrhea. Also, age, sex, and size of child at birth were statistically associated with bloody diarrhea in children. Children age 12–59 months were 1.83–2.78 (95% CI: 1.60, 3.38) times more likely to suffer from bloody diarrhea compared with children age 0–11 months. Male children were 1.11 (AOR: 1.11; 95% CI: 1.01, 1.23) times as likely to suffer from bloody diarrhea as female children. Children who were of average- or large- size at birth were 1.17 (AOR: 1.17; 95% CI: 1.02, 1.35) times as likely to suffer from bloody diarrhea as children who were of small size at birth. Further, the literacy status of the mother was statistically associated with bloody diarrhea in children. Children of illiterate mothers were 1.24 (AOR: 1.24; 95% CI: 1.10, 1.39) times as likely to suffer from bloody diarrhea as children of literate mothers. Moreover, the children residing in rural areas were 1.15 (AOR: 1.15; 95% CI: 1.01, 1.33) times as likely to suffer from bloody diarrhea as children residing in urban areas.

The results of multinomial logistic regression showing the determinants of treatment of bloody diarrhea in children are shown in Table 4. The first place of treatment sought for bloody diarrhea was statistically associated with inadequate and adequate treatment in children. Public health facility was 1.75 times (AOR: 1.17; 95% CI: 1.16, 2.64) as likely as other health facility to be used as the first place to receive inadequate treatment for bloody diarrhea compared to no treatment. Also, the public health facility was 3.13 times (AOR: 3.13; 95% CI: 1.88, 5.23) as likely as other health facility to be used as the first place to receive adequate treatment for bloody diarrhea compared with no treatment. The age of the child was statistically associated with adequate treatment in children. The children age 35–59 months were 1.70 times (AOR: 1.70; 95% CI: 1.13–2.58) as likely as children below 18 months to receive adequate treatment for bloody diarrhea compared to no treatment. Further, mother’s literacy status was statistically associated with inadequate treatment in children. The children of literate mothers were 1.33 times (AOR: 1.33; 95% CI: 1.01–1.76) as likely as children of non-literate mothers to receive inadequate treatment for bloody diarrhea compared with no treatment.

**Table 4:** Results of multinomial logistic regression showing the socioeconomic, demographic and residence-related determinants of treatment of bloody diarrhea in children below five years, India, 2015–16 (N = 1545).

Socioeconomic, demographic and residence related determinants	Inadequate treatment	Adequate treatment
	AOR (95% CI)	AOR (95% CI)
**First place of treatment sought for bloody diarrhea**		
Other	Ref.	Ref.
Public	**1.75 (1.16–2.64)**	**3.13 (1.88–5.23)**
Private	1.03 (0.72–1.47)	0.74 (0.46–1.22)
**Days within treatment sought after onset of bloody diarrhea**		
After two days	Ref.	Ref.
Same day	1.21 (0.87–1.68)	1.47 (0.97–2.24)
Second day	1.04 (0.78–1.38)	1.34 (0.92–1.95)
**Age of child (in months)**		
Below 18 months	Ref.	Ref.
18–35 months	1.30 (0.98–1.71)	1.41 (0.98–2.02)
36–59 months	1.39 (0.99–1.93)	**1.70 (1.13–2.58)**
**Sex of child**		
Male	Ref.	Ref.
Female	0.87 (0.68–1.11)	1.06 (0.77–1.45)
**Literacy status of mother**		
Non-literate	Ref.	Ref.
Literate	**1.33 (1.01–1.76)**	1.11 (0.77–1.59)
**Mother exposed to mass media**		
Unexposed	Ref.	Ref.
Exposed	1.06 (0.78–1.43)	**2.38 (1.55–3.66)**
**Religion**		
Hindu	Ref.	Ref.
Other	0.98 (0.73-.1.32)	**1.45 (1.01–2.08)**
**Caste/Tribe**		
Other	Ref.	Ref.
SC and ST	1.21 (0.94–1.56)	1.36 (0.98–1.89)
**Household wealth status**		
Poor	Ref.	Ref.
Middle	1.11 (0.83–1.51)	1.26 (0.86–1.86)
Rich	1.43 (0.95–2.16)	**1.93 (1.18–3.16)**
**Place of Residence**		
Rural	Ref.	Ref.
Urban	**1.50 (1.04–2.15)**	1.35 (0.87–2.10)

Abbreviations: AOR, adjusted odds ratio; CI, confidence interval; Ref, reference; SC, scheduled caste; ST, scheduled tribe.

Bold AOR indicates significant finding at p-value < 0.05.

The mother’s exposure to mass media, religion, and household wealth status were also statistically associated with adequate treatment in children. The children of mothers exposed to mass media were 2.38 times (AOR: 2.38; 95% CI: 1.55–3.66) as likely as children of mothers not exposed to mass media to receive adequate treatment compared to no treatment. The children belonging to other religion were 1.45 times (AOR: 1.45; 95% CI: 1.01–2.08) as likely as children belonging to Hindu religion to receive adequate treatment compared with no treatment. The children belonging to rich households were 1.93 times (AOR: 1.93; 95% CI: 1.18–3.16) as likely as children belonging to poor households to receive adequate treatment compared to no treatment. Further, the place of residence was statistically associated with inadequate treatment in children. The children residing in urban areas were 1.50 times (AOR: 1.50; 95% CI: 1.04–2.15) as likely as children residing in rural areas to receive inadequate treatment compared with no treatment.

## Discussion

To the best of our knowledge, our study is the first to investigate the risk factors and management of bloody diarrhea in the youngest children under five years using a large-scale population-based nationally representative data in India. The mean age of children who suffered from bloody diarrhea was higher than the mean age of children who suffered from watery diarrhea in our study. Our finding is similar to that of a study in Turkey, where children having bloody diarrhea had a higher mean age than children having watery diarrhea [13]. We found a little less than one-tenth children with diarrhea suffered from bloody diarrhea in the last two-weeks of the survey. One of the major findings of our study reveals a statistical association between household environmental risk factors, such as unsafe disposal of children’s stools and a dedicated place with water for washing hands in the household, and occurrence of bloody diarrhea. This finding is consistent with previous two studies from Kenya which reported that hand washing after defecating and disposing of child’s stools were protective practices against bloody diarrhea in children [14, 15]. Also, a recent study from India concluded that unsafe disposal of children’s stools in the neighborhood puts children at a higher risk of watery diarrhea even if their stools were disposed off safely [23]. The invasive bacterial infection is the most common cause of bloody diarrhea in children in underdeveloped and developing countries. It transfers through stools of infected children via direct fecal-oral route [5]. Hence, unsafe disposal of children’s stools and not washing hands after defecation make children more susceptible to fecal pathogens causing bloody diarrhea.

We found that the bottle fed children were more likely to have bloody diarrhea. A few other studies have also observed that bottle feeding was associated with both bloody diarrhea and watery diarrhea in children [7, 36]. In contrary to previous studies, we did not find the association between bloody diarrhea and type of toilet facility, drinking water source and treatment and initiation of breastfeeding after birth. These studies have reported the risk of bloody/watery diarrhea was higher among children belonging to the households with unimproved toilet facilities, using untreated and unimproved drinking water and received breastfeeding after one hour of birth [14, 15, 27]. Further, our study reports that the risk of bloody diarrhea increases with increase in child age and this finding is similar to a study from Iraq [7]. Strikingly, it is opposite to the case of watery diarrhea where the risk decreases with increase in age in children [23, 37]. Moreover, we found that the male children were at a higher risk of bloody diarrhea and a few other studies too reported that male children were more likely to suffer from watery diarrhea [23, 28, 36]. The findings further reports that the risk of bloody diarrhea is higher among children born with average and larger size and it is contrary to previous studies indicating higher risk of watery diarrhea among children born with smaller than average size [23, 28, 38]. Furthermore, the mother’s non-literacy is found to be a significant risk factor of bloody diarrhea in our study. This finding is supported by another study which concluded that children of non-literate mothers had a higher risk of bloody diarrhea in Iraq [7]. Talking about other socioeconomic factors, we found that bloody diarrhea was significantly higher among children belonging to other religion (minority), STs and SCs (marginalized social groups), and those staying in rural areas. These findings are also consistent with studies from different settings [7, 23, 28, 29].

Our study also brings to the forefront the prevalence of the treatment of bloody diarrhea in the Indian children. We found a little more than one-fourth of the children did not receive any treatment for bloody diarrhea even after visiting health facility while only a little less than one-fifth children received adequate treatment. The findings further suggest that private health facility was the first line of contact for the majority of children, but most of the children received adequate treatment of bloody diarrhea from a public health facility. In connection, the Healthcare utilization and attitudes surveys (HUASs) (under GEMS) found about 27% children did not receive any treatment for diarrhea while licensed practitioner was the first line of contact for treatment in India [19]. A study from Guatemala revealed that no medical help was sought for 7% of the children and the local store was the first source for treatment for diarrhea [39].

Likewise, the finding that the children are more likely to receive any treatment of bloody diarrhea from public health facility is consistent with two other studies suggesting that caregivers of children preferred government/public health facility over private and other health facilities due to easy access and minimal cost in Ethiopia [30, 40]. Another study also reported that access to a health facility was a major barrier for timely treatment seeking for diarrhea in India [35]. We found the age of child as a significant predictor for treatment of bloody diarrhea and this finding is also similar to two previous studies from Ethiopia [30, 41]. Like previous studies, our study too observed that children of literate mothers and mothers exposed to mass media are more likely to receive some treatment for bloody diarrhea. The previous studies suggested that the treatment seeking was delayed among the illiterate mothers compared to literate mothers in India and Ethiopia [30, 35]. However, one study reported that illiterate mothers were more likely to seek informal care for diarrhea in children in Ethiopia [40]. Few studies also indicated that maternal access to electronic media (television and radio) and newspaper significantly influenced timely and formal health care seeking and use of oral rehydration and zinc for diarrhea in different settings [32, 40, 42]. We found that the religion is one of the important socioeconomic determinants of treatment of bloody diarrhea and a qualitative study from Kenya, Nigeria, and Niger support this finding. The study reported that many times, religious beliefs and authorities prohibited treatment seeking for diarrhea in children from health facilities [33]. Likewise, our findings suggest that children belonging to rich households and residing in urban areas are more likely to receive any treatment. This finding is also similar to a few previous studies indicating that children from rich households, and urban areas receive timely, formal treatment and oral rehydration and zinc in India and Ethiopia [32, 35, 40].

A recent NFHS-4 report suggested a 15 percent decline in the unsafe disposal of children’s stools and a 20 percent increase in households accessing improved toilet facilities during the last decade in India [25]. In 2014, the Government of India launched the Swachh Bharat Mission (SBM) to achieve a clean and open defecation free (ODF) India by October 2019. According to recent reports, the rural sanitation coverage increased from 42 percent in October 2014 to 64 percent in May 2017 under SBM [43]. It must be noted that the effective disposal of children’s stools is one of the key indicators for declaring ODF under SBM [44]. However, we found that stools of nearly 35 percent of the children are disposed off unsafely, even if households have access to improved toilet facilities. Moreover, the unsafe disposal of children’s stools is common in rich and urban households too. A recent study in rural Odisha reported that an increase in sanitation coverage did not have much impact on the safe disposal of children’s stools [45].

Washing hands is an effective, feasible and cost-effective intervention for preventing diarrheal diseases. A systematic review of seventeen studies reported that washing hands could reduce the risk of childhood diarrhea by 47 percent [46, 47]. Thus, washing hands has received much attention globally, and since 2008, every year, 15^th^ October is observed as Global Handwashing Day (GHD). India is not far behind and observes GHD in partnership with the Ministry of Rural Development and Water Supply and Sanitation and State Education Departments [48]. The Government of India is also instrumental in promoting washing hands among children and caregivers through various activities and programs. Interestingly, NFHS-4, the first time in the NFHS series provided information on the provision of a place for washing hands in the households [25]. We found that 17 percent of the children belonged to households with neither a place nor water to wash hands; 85 percent of those children reside in rural areas, and 60 percent belong to poor wealth status.

Dehydration is the most common complication of bloody diarrhea in children. The WHO guidelines for the management of common childhood illnesses recommend that children should receive intravenous/oral rehydration, food-based fluids (soup, rice water, and yogurt drinks) immediately and clean water at home and in the health facility [49]. But oral rehydration is not a sufficient treatment for some children with bloody diarrhea because they may have a bacterial or parasitic infection that requires antimicrobial treatment [50]. Hence, IMCI guidelines recommend that children having bloody diarrhea must immediately be referred to health facility/center and receive antibiotics, fluids, feeding, and follow up [18]. Surprisingly, our data show that about 17 percent of children did not visit any health facility for the treatment of bloody diarrhea. Likewise, 28 percent of the children did not receive any treatment on visiting any health facility. It is noteworthy that most of these children reside in rural areas, belong to uneducated mothers and poor households. Strikingly, India is one of the fewest countries in the world where 5–10% of diarrheal deaths in children are attributable to bloody diarrhea [8]. Hence, awareness about the immediate treatment of bloody diarrhea among mothers/caregivers could reduce the diarrheal deaths in children.

Our findings have important policy implications and lend support to SBM for effective implementation and increasing sanitation coverage in India. But SBM should expand its focus to safe disposal of children’s stool beyond toilet promotion and ODF status. It aims to spend 8 percent to 15 percent of the annual budget on information, education, and communication (IEC), behavior change communication (BCC) activities and public awareness [43, 51]. SBM needs to create awareness on the ill-effects of unsafe disposal of children’s stools and its linkages with diarrhea and bloody diarrhea. The SBM needs to undertake massive public campaigns on the usage of toilets for safe disposal of children’s stools.

Our findings also suggest that the dedicated place and water for washing hands is a must to reduce children’s susceptibility to bloody diarrhea. The National Rural Drinking Water Program (NRDWP) must act together with SBM to ensure availability of water, especially in poor households. The availability of adequate water for sanitation activities needs to be given a priority under NRDWP and SBM. NRDWP and SBM need to engage Accredited Social Health Activists (ASHA), Anganwadi workers (AWW), Swachhta facilitators, Village Health, Sanitation, and Nutrition Committee (VHSNC) members and Panchayat members need to create awareness about the benefits of safe disposal of children’s stools and increase knowledge, attitudes, and practices (KAP) about hand hygiene in the context of child health. Health workers need to educate mothers to identify the signs of diarrhea in general and bloody diarrhea in particular. The workers should advise mothers to start oral rehydration immediately at home and visit a health facility/center as early as possible in case of bloody diarrhea.

### Limitations

Our study also has a few limitations. First, we used cross-sectional data to determine the prevalence of bloody diarrhea. Second, our estimates of bloody diarrhea are based on mother’s 14-day recall period. But, it may be noted that all DHS and UNICEF sponsored surveys are cross-sectional and use two-week recall to determine the prevalence of childhood illnesses. Third, our analysis includes only the youngest child because data on children’s stool disposal and initiation of breastfeeding were not collected for other children in birth history. However, there are a few studies on similar lines that have included only youngest child from birth history and results have been generalized to all under-five children [23, 28]. Fourth, we used children’s birth size as a proxy for birth weight because birth weight information was not available for 22 percent of the children in NFHS-4 [25]. Studies have shown that birth size is a good indicator of birth weight in settings having a dearth of accurate and reliable data on birth weight [52–54]. Fifth, we used the place for washing hands in the households as a proxy for mother/children’s handwashing practice. The dedicated handwashing place in the households does not ensure that mothers/children wash their hands after defecation. It can also be argued that mothers and children can practice handwashing even if the household does not have a dedicated place for washing hands. Sixth, we analyzed ‘treatment of bloody diarrhea (no/inadequate/adequate)’ using ‘first place of treatment sought for bloody diarrhea (public, private and other) and not with the type of health care provider (qualified/unqualified). Hence it can be taken as one of the limitations which requires further research. Seventh, some studies also reported that prior use of antibiotics could cause bloody diarrhea in children, however, our data did not give us the opportunity to check for this possibility [55, 56].

## Conclusion

To conclude, our study brings to the forefront the prevalence of bloody diarrhea and its management and associated factors in the youngest children below age five in India. We found that unsafe disposal of those children’s stools poses a serious threat to children. The absence of a dedicated place and water for washing hands in households puts the youngest children at a higher risk of bloody diarrhea. Despite various child health programs and interventions, a significant proportion of children neither visit any health facility for treatment of bloody diarrhea nor receive any treatment. In light of our findings, we suggest that SBM extend the focus on safe disposal of children’s stools and hand hygiene along with toilet promotion and ODF status. NRDWP and SBM should have an integrated approach and ensure that households, particularly, rural and poor households have an adequate supply of water for drinking and sanitation purposes. Frontline health workers, ASHA, AWW, and Swachhta facilitators should conduct regular IEC and BCC activities related to KAP about hand washing. Moreover, they can undertake regular spot check evaluation for a dedicated place in each household to wash hands and hand hygiene practices during home visits. Last but not the least, mothers must be advised to use oral rehydration immediately at home and refer a child with bloody diarrhea to a health facility/center as early as possible.

## Supporting information

S1 File S1_Supporting_Information_Variable_Description_File.docx. (DOCX)
