# Supplementary material for: Epidemiological investigation and management of bloody diarrhea among children in India
Source: PLoS One. 2019 Sep 13;14(9):e0222208. doi: 10.1371/journal.pone.0222208 (PMC6743764; doi:10.1371/journal.pone.0222208)
Supplement: S1 File — (DOCX) [file pone.0222208.s001.docx]

**S1 Appendix: Variable description file**

| **Sr. No.** | **Control variables** | **Category** |
| --- | --- | --- |
| **1** | **Type of toilet facility** | 1. **Improved** (Flush/pour flush to piped sewer system; Flush/pour flush to septic tank; Flush/pour flush to pit latrine; Ventilated improved pit (VIP) latrine/biogas latrine; Pit latrine with slab; Twin pit, composting toilet)  2. **Unimproved** (Flush/pour flush not to sewer/septic  tank/pit latrine; Pit latrine without slab/open pit; Dry toilet; Other; No facility/uses bush/field) |
| **2** | **Drinking water source** | 1. **Improved** (Piped water into dwelling or to yard/plot; Public tap/standpipe; Tube well or borehole; Protected dug well; Protected spring; Rain water; Community RO plant)  2. **Unimproved** (Unprotected dug well; Unprotected spring; Tanker truck/cart with small tank; Surface water; Bottled water; Other) |
| **3** | **Drinking water treatment** | 1. **Treated** (Boil; Use alum; Add bleach/chlorine; Strain through cloth; Use ceramic, sand, or other water filter; Use electric purifier; Allow water to stand and settle; Other)  2. **Untreated** (No treatment) |
| **4** | **Children’s stool disposal** | 1. **Safe** (Used toilet/latrine; Put/rinsed into toilet/latrine; Put/rinsed into drain/ditch; Buried)  2. **Unsafe** (Thrown in garbage; Left in the open; Other; Don’t know) |
| **5** | **Presence of handwashing place** | 1. **With water and cleansing agent** (place with water, and any cleansing agent (soap/detergent (bar, liquid, powder, paste), ash, mud, and sand)  2. **With water only** (place with water but without any cleansing agent)  3. **Neither handwashing place nor water for hand wash** |
| **6** | **Initiation of breastfeeding after birth** | 1. **Within 1 hour** (Immediately; Within one hour)  2. **Within 1-24 hour**  3. **After 24 hours** |
| **7** | **Bottle feeding** | 1. **Yes** (Drank from bottle with nipple yesterday/last night)  2. **No** |
| **8** | **Age of child (in months)** | 1. Below 12 months (0-11 months)  2. 12-23 months  3. 24-35 months  4. 36-47 months  5. 48-59 months |
| **9** | **Sex of child** | 1. Male  2. Female |
| **10** | **Size of child at birth** | 1. **Average or larger** (Very larger; Larger than average; Average)  2. **Smaller than average** (Smaller than average; Very small; Don’t know) |
| **11** | **Literacy status of mother** | 1. **Non-literate** (Did not complete a single year of education)  2. **Literate** (Completed at least 1 year of education) |
| **12** | **Mother exposed to mass media** | 1. **Unexposed** (Neither watch TV nor listen radio nor read magazine)  2. **Exposed** (Either watch TV or listen radio or read magazine) |
| **13** | **First place of treatment sought for bloody diarrhea** | 1. **Public** (Govt./Municipal Hospital; Vaidya/Hakim/Homeopath (AYUSH); Govt. Dispensary; UHC/UHP/UFWC; CHC/Rural Hosp./Block PHC; PHC/Additional PHC; Sub-Center/ANM; Govt. Mobile Clinic; Camp; Anganwadi/ICDS Center; ASHA, Other Public Health)  **2. Private** (Hospital; Pharmacy/Drug Store; Doctor/Clinic; Paramedic; Vaidya/Hakim/Homeopath (AYUSH); Other Private Health)  **3. Other** (NGO or Trust Hospital/Clinic; Shop; Traditional Healer; Friend/Relative; Other) |
| **14** | **Days within treatment sought after onset of bloody diarrhea** | 1. Same day  2. Second day  3. After two days |
| **15** | **Religion** | 1. Hindu  2. Other (Muslim; Christian; Sikh; Buddhist/Neo-Buddhist; Jain; Jewish; Parsi/Zoroastrian; No religion; Other) |
| **16** | **Caste** | 1. SC and ST (Scheduled Caste; Scheduled Tribe)  3. Other (OBC; None of them) |
| **17** | **Wealth status^1^** | 1. Poor  2. Middle  3. Rich |
| **18** | **Place of residence** | 1. Urban  2. Rural |

^1^ determined using the information on household assets: Mattress, Pressure Cooker, Chair, Cot or Bed, Table, Electric Fan, Radio, Transistor, Television, Sewing Machine, Mobile Telephone, Landline Telephone, Internet, Computer, Refrigerator, Air Conditioner/Cooler; Washing Machine, Watch/Clock, Water Pump, Thresher, Tractor, Bicycle, Motorcycle or Scooter, Animal Drawn Cart, Car, Bank Account, Electricity, House Ownership, Persons Per Room, Roof Material, Wall Material, Floor Material, and Cooking Fuel.
